# Recent Advances in the Emerging Therapeutic Strategies for Diabetic Kidney Diseases

**DOI:** 10.3390/ijms231810882

**Published:** 2022-09-17

**Authors:** Wei Huang, Yi-Yuan Chen, Zi-Qi Li, Fang-Fang He, Chun Zhang

**Affiliations:** Department of Nephrology, Union Hospital, Tongji Medical College, Huazhong University of Science and Technology, Wuhan 430022, China

**Keywords:** diabetic kidney disease, SGLT2 inhibitors, fibrosis, inflammation, therapeutics

## Abstract

Diabetic kidney disease (DKD) is one of the most common causes of end-stage renal disease worldwide. The treatment of DKD is strongly associated with clinical outcomes in patients with diabetes mellitus. Traditional therapeutic strategies focus on the control of major risk factors, such as blood glucose, blood lipids, and blood pressure. Renin–angiotensin–aldosterone system inhibitors have been the main therapeutic measures in the past, but the emergence of sodium–glucose cotransporter 2 inhibitors, incretin mimetics, and endothelin-1 receptor antagonists has provided more options for the management of DKD. Simultaneously, with advances in research on the pathogenesis of DKD, some new therapies targeting renal inflammation, fibrosis, and oxidative stress have gradually entered clinical application. In addition, some recently discovered therapeutic targets and signaling pathways, mainly in preclinical and early clinical trial stages, are expected to provide benefits for patients with DKD in the future. This review summarizes the traditional treatments and emerging management options for DKD, demonstrating recent advances in the therapeutic strategies for DKD.

## 1. Introduction

Diabetic kidney disease (DKD) is one of the most common and serious complications of diabetes mellitus (DM) and remains the leading cause of end-stage renal disease (ESRD) in Western countries [1]. The global prevalence of DM is rapidly increasing, and around 35–40% of patients with type 1 DM (T1DM) or type 2 DM (T2DM) develop DKD, which results in devastating clinical outcomes [2]. The grave burden of DM has made the treatment of DKD an urgent problem to be solved.

Although there were no specific drugs with the exception of renin–angiotensin–aldosterone system (RAAS) inhibitors for a long time, the therapeutic strategies for DKD have advanced significantly over the last decade [3]. With clinical trials reporting positive results, a variety of drugs that have renoprotective effects are emerging, including sodium–glucose cotransporter 2 (SGLT2) inhibitors, glucagon-like peptide-1 receptor (GLP-1R) agonists, and dipeptidyl peptidase-4 (DPP-4) inhibitors [4]. Furthermore, major advances have been made in understanding the pathogenesis of DKD, and the roles of oxidative stress, renal fibrosis, and inflammation have been emphasized [5]. As such, these pathways present potential targets for the treatment of DKD. This review will focus on the current and emerging therapeutic strategies for DKD and summarize recent advances in DKD management.

## 2. Lifestyle Improvements

Lifestyle intervention is an essential component of the strategy for managing DM and its complications. For all patients with DM, lifestyle modifications, including weight loss, smoking cessation, adequate physical exercise, and restriction on sodium and protein intake, are crucial to preventing the onset of DKD and other adverse events [6].

Obesity is a powerful risk factor for albuminuria, and DKD is more prevalent in patients with DM who are obese [7]. Obesity has adverse effects on blood glucose, blood lipids, and blood pressure, which are closely associated with DKD [7]. Furthermore, obesity directly influences the kidneys by changing glomerular hemodynamics and increasing mechanical compression [8]. One clinical study showed the beneficial effects of weight loss on reducing the incidence of albuminuria and progressive kidney diseases in patients with DM following a low-calorie diet or bariatric surgery [9].

Smoking is an independent risk factor for the occurrence and development of DKD [10]. A meta-analysis of 41,271 subjects with T2DM revealed that smoking was one of the most significant baseline indicators related to renal events, and the incidence of DKD increased by almost 50% in subjects who smoked [11]. The Kidney Disease: Improving Global Outcomes (KDIGO) guidelines suggest that patients with established DKD or patients who are at risk of DKD should avoid smoking [12].

Clinical data of patients with T1DM or T2DM have demonstrated that regular physical activity improves insulin sensitivity and cardiovascular outcomes [13]. One animal experiment has also shown that albuminuria is reduced in rats with DM by increasing exercise [14]. It is recommended that patients with DM perform 150 min of moderate exercise weekly, but for patients with overt nephropathy, carefully monitoring blood pressure during exercise is necessary [15].

Sodium restriction has been demonstrated to afford a potent benefit with respect to albuminuria in patients with DM [16]. Standard protein restriction (<0.8 g per kg of body weight per day) is not generally recommended, as it is difficult to achieve clinically and may increase the risk of malnutrition and bone remodeling [17]. Thus, a more reasonable protein intake needs to be established in future studies.

In addition, recent studies have illustrated that the influence of lifestyle on the occurrence and development of certain metabolic diseases may begin as early as embryonic stage, and the fetal programming hypothesis has been derived [18,19]. Fetal programming proposes that the fetus develops adaptations to its growth environment that persist after birth and may lead to metabolic diseases in adulthood [18]. For example, previous studies have shown that maternal malnutrition, calorie restriction, and a high-fat diet can adversely affect fetal kidney production, predisposing offspring to kidney diseases [19,20]. Mothers with DKD have higher rates of preterm birth, and their babies are more likely to have congenital abnormalities such as congenital kidney and urinary tract abnormalities, than the babies of mothers with DM without kidney diseases [21]. Paternal obesity can result in higher triglyceride concentrations in the kidneys of offspring, as well as increasing the risk of tubular damage [22]. One study analyzed the effects of feeding male rats a high-fat, high-sugar, and high-salt diet over two generations (F0 and F1) on the renal function of their offspring using the estimated glomerular filtration rate (eGFR) and the urinary albumin-to-creatinine ratio (UACR) as indicators of kidney damage. The eGFR and UACR were not obviously changed in F1 offspring, whereas F2 female offspring showed a significant decrease in eGFR and a significant increase in UACR [23]. The underlying molecular mechanism of fetal programming may be mediated by epigenetic modification. The lifestyle habits and physiological states of the parents lead to early epigenetic changes in spermatogenesis, which in turn lead to changes in target organs and the emergence of specific phenotypes in offspring [24]. DNA methylation is an important epigenetic modification that plays a key role in the development of DM and DKD and is closely related to renal fibrosis [25]. This suggests treatment targeting DNA methylation in parents with a genetic risk of DKD during fetal development as a novel lifestyle intervention.

## 3. Pharmacological Therapies

### 3.1. Glycemic Control

Poor glycemic control is an independent predictor of albuminuria and ESRD progression in patients with DM [26]. Several clinical trials have demonstrated that diabetic complications, including renal disease, are mainly observed in the context of elevated glucose concentrations, and in addition to chronic hyperglycemia, even transient increases in blood glucose may have long-term influence on the progression of DKD [27,28]. Thus, in patients with classic DKD, standard therapy still focuses on glycemic control, and the effects of intensive glycemic control on preventing DKD during early DM have been validated in some large clinical trials [29,30,31].

The importance of intensive glycemic control in reducing albuminuria and improving the reduction in eGFR were initially confirmed in the Diabetes Control and Complications Trial (DCCT) and the Epidemiology of Diabetes Interventions and Complications (EDIC) study [29,30]. In the subsequent United Kingdom Prospective Diabetes Study (UKPDS), although there was no significant difference in doubling of serum creatinine (SCr) compared with placebo, a reduced rate of development of albuminuria was observed in the intensive glycemic control group [31]. Furthermore, one observational study further validated that intensive glycemic control could reduce the risk of developing ESRD, especially in patients with preserved renal function [32].

However, a retrospective analysis demonstrated that the renoprotective effect of intensive glycemic control was predominantly observed in patients with early DM and subjects without kidney diseases [33]. For patients with long-term DM or patients with kidney diseases, intensive glycemic control did not prevent the progression of DKD. In fact, it might incur a great risk of hypoglycemia and higher cardiovascular mortality or all-cause mortality, especially in patients with a therapeutic goal of glycosylated hemoglobin (HbA1c) below 6.9% [34]. Each class of glucose-lowering agent has side effects and suitable populations. Although the National Kidney Foundation Disease Outcomes Quality Initiative (KDOQI) guidelines suggest keeping HbA1c levels ≤7.0% in patients with DM, for individuals with longstanding DM, cardionephric complications, and a limited life expectancy, the target HbA1c level can be adjusted to >7.0%. These findings suggest that glycemic control in patients with DM should be individualized according to the patient’s sensitivity to hypoglycemia and the potential cardionephric disease status.

### 3.2. Blood Lipid Control

Hyperlipidemia is another risk factor for DKD progression, especially increases in triglycerides, low-density lipoprotein cholesterol (LDL-C), and apolipoprotein-B-100, which may increase the incidence of cardiovascular events in patients with DM with persistent albuminuria [35]. Thus, the KDOQI guidelines recommend that patients with DKD receive statin therapy to prevent cardiovascular events [36].

The renoprotective effects of statin-based treatments remain to be demonstrated. The Anglo-Danish-Dutch Study of Intensive Treatment in People with Screen Detected Diabetes in Primary Care (ADDITION-Europe) claimed no obvious renoprotective effect of statins in patients with DM [37], but the Prospective Evaluation of Proteinuria and Renal Function in Diabetic Patients with Progressive Renal Disease trial (PLANET I trial) revealed that atorvastatin reduced renal events and albuminuria in patients with DKD [38]. The cause of this heterogeneity may be that subjects in the PLANET I trial accepted treatment with atorvastatin and RAAS inhibitors simultaneously. On the contrary, fibrates, such as fenofibrate, were clearly proven to protect the kidneys and demonstrated a reduction in albuminuria in patients with DM in the Fenofibrate Intervention and Event Lowering in Diabetes (FIELD) trial [39]. However, the fact that fenofibrate may increase SCr limits its application in populations with established DKD.

### 3.3. Blood Pressure Control

Hypertension is an important factor associated with DKD progression [40]. One animal model has confirmed that controlling blood pressure in rats with DM can reduce the onset of albuminuria and glomerulosclerosis [41]. One randomized controlled trial designed to test the effects of blood pressure control on cardiovascular events in patients with DM demonstrated that systolic blood pressure was linearly associated with an increased incidence of myocardial infarction, stroke, and DKD [42]. One clinical study of patients with T2DM with hypertension found that blood pressure variability was related to albuminuria and HbA1c level, and systolic blood pressure variability was a predictor of the degree of kidney damage [43]. One recent observational study evaluating the effect of changes in blood pressure on renal function showed that a 5 mmHg increase in blood pressure variability was associated with a 5% increased risk of albuminuria [44]. These studies suggest that controlling blood pressure and minimizing the variability of blood pressure might delay the progression of DKD.

In addition to increasing the risk of cardiovascular events, hypertension can activate the production of multiple cytokines, chemokines, and growth factors through hemodynamic mechanisms, leading to renal inflammation and fibrosis [45]. Glomerular hypertension in patients with DM can also directly cause podocyte detachment by increasing mechanical stress [46]. The treatment of hypertension could alleviate renal inflammation and fibrosis and mitigate podocyte injury.

Hyperglycemia can lead to glomerular hypertension through multiple pathways, such as dilating afferent arterioles by releasing vasoactive substances and tubuloglomerular feedback, and constricting efferent arterioles by increasing local angiotensin II (Ang II) levels [47]. The RAAS is one of the best-studied mechanisms of blood pressure regulation, and therapies targeting the RAAS occupy a central position in the field of DKD treatment.

RAAS inhibitors, which mainly include angiotensin-converting enzyme inhibitors (ACEIs) and angiotensin receptor blockers (ARBs), have been used to treat DKD for several decades. RAAS inhibitors have been widely demonstrated to be effective in protecting kidney function in several landmark studies [48,49,50]. The captopril study showed that captopril had benefits in patients with T1DM with overt albuminuria [48]. The Renal and Cardiovascular Outcomes in Patients with Type 2 Diabetes and Nephropathy Study (RENAAL) and the Irbesartan Diabetic Nephropathy Trial (IDNT) study, respectively, illustrated the benefits of losartan and irbesartan in patients with T2DM [49,50].

The renoprotective effect of RAAS inhibitors was initially considered to be achieved by controlling blood pressure and was not recommended for use in patients with DKD without hypertension [51]. However, RAAS inhibitors have been reported to exert many nonhemodynamic effects on the kidneys. For example, Ang II, the core member of the RAAS, can increase glomerular capillary pressure, stimulate renal cell proliferation, promote extracellular matrix (ECM) synthesis, and cause macrophage infiltration to participate in fibrosis and inflammation, which contribute to the development of kidney injury [52,53]. RAAS inhibitors could partially reverse the above changes to alleviate fibrosis and inflammation in patients with DKD. Furthermore, increasing interest in podocyte biology has suggested that blocking the RAAS may directly reverse the damaged podocyte structure in patients with DM [54]. The evidence suggests that RAAS inhibitors have benefits beyond their blood-controlling effect.

### 3.4. Aldosterone Antagonists

Since the combination of ACEI and ARB did not provide more beneficial effects than monotherapy and instead increased the risk of hyperkalemia and acute kidney injury (AKI), the enthusiasm for enhanced RAAS blockade shifted to combining mineralocorticoid receptor antagonists (MRAs) with RAAS inhibitors [55]. Aldosterone, a mineralocorticoid hormone produced via the activation of the mineralocorticoid receptor (MR) by AngII, is involved in the pathogenesis of DKD [56]. Treatment with ACEI or ARB can only partially suppress aldosterone, but the addition of MRAs may offer additional renoprotective effects [57].

Spironolactone and eplerenone are the first studied MRAs. Animal studies have demonstrated that spironolactone could improve glomerulosclerosis in rats with DM, and eplerenone could decrease intraglomerular pressure and albuminuria in mice with DM [58,59]. Eplerenone has a synergistic effect with enalapril of preventing albuminuria and glomerulosclerosis in rats with DM [60]. One meta-analysis confirmed that spironolactone combined with RAAS inhibitors could significantly reduce albuminuria and UACR in patients with DKD [61]. However, the high risk of hyperkalemia limits the application of the two drugs [62]. The Eplerenone in Mild Patients Hospitalization and Survival Study in Heart Failure (EMPHASIS-HF) study demonstrated the beneficial effects of eplerenone for patients with heart failure, but its post hoc analysis found that patients with reduced eGFR had a higher incidence of hyperkalemia even when they received lower doses of eplerenone [63,64]. Finerenone is a novel MRA with high selectivity for MR and fewer side effects [65]. One study with 96 patients with T2DM demonstrated that the combination of finerenone and RAAS inhibitors improved albuminuria without increasing serum potassium levels [66]. Two subsequent large clinical trials with patients with DM and chronic kidney diseases, the Effect of Finerenone on Chronic Kidney Disease Outcomes in Type 2 Diabetes (FIDELIO) study and the Cardiovascular Events with Finerenone in Kidney Disease and Type 2 Diabetes (FIGARO) study, investigated the long-term effects of finerenone on kidney and cardiovascular outcomes and showed that finerenone had a significant cardio-renal protective effect, especially for patients with severe kidney disease [67,68]. Although finerenone carried a risk of hyperkalemia, no fatal cases have been reported and discontinuation rates due to hyperkalemia were low. Finerenone has now been approved for DKD indications. Additionally, one recent study analyzed the effects of empagliflozin, a SGLT2 inhibitor, in patients with heart failure treated with MRAs. The results showed that patients receiving empagliflozin had lower rates of hyperkalemia and withdrawal of MRAs compared with placebo, suggesting that combination therapy may be an effective approach to improving the safety of MRAs [69]. Major clinical studies related to aldosterone antagonists are summarized in Table 1.

The underlying mechanism of the renoprotective function of MRAs is still not fully understood, and some preclinical studies have shown that it may be related to the reduction of renal neutrophil gelatinase-associated lipocalin (NGAL) and the inhibition of Rac1 [70,71]. However, the beneficial effects of combined therapy with MRAs and RAAS inhibitors in patients with DKD have been confirmed. In the future, combination therapy may replace traditional RAAS blockade as routine prevention and management in patients with DM with risk factors for DKD.

### 3.5. Diuretics

As mentioned above, the antialbuminuric effects of aldosterone antagonists have been established in clinical studies, and while the efficacy of other diuretics for DKD has been less well-studied, evidence suggests that they may have similar antialbuminuric properties.

Several studies have shown that thiazide diuretics, such as hydrochlorothiazide and losalidone, have obvious albuminuria-reducing effects in patients with DKD, possibly by reducing intraglomerular pressure, but there are also results supporting that their renoprotective effects may be independent of their antihypertensive effects [72,73,74]. A low-sodium diet clearly enhances the antialbuminuric effects of RAAS inhibitors, but this is often difficult to achieve clinically, although the natriuretic effect of diuretics may address this issue. One study involving 45 patients with DM evaluated effects of sodium restriction and hydrochlorothiazide on albuminuria. The experimental group received hydrochlorothiazide or sodium restriction, while the control group received placebo or a regular sodium diet. The results showed that both sodium restriction and hydrochlorothiazide significantly reduced albuminuria [75]. Another study involving 34 nondiabetic patients showed that RAAS blockers combined with sodium restriction and RAAS blockers combined with diuretic therapy were nearly equally as effective in reducing albuminuria and blood pressure [76]. These results suggest that the addition of thiazides may be a good option for patients with DKD who cannot achieve a low-sodium diet.

Other diuretics have also been reported to have renoprotective effects. One study showed that the addition of furosemide on the basis of half-dose RAAS inhibitor had a better antialbuminuric efficacy than full-dose RAAS inhibitor therapy [77]. Thiazide diuretics combined with loop diuretics have been shown to improve eGFR in patients with DKD [78]. This effect may be partly explained by the powerful hypotensive effect of loop diuretics. However, there is currently no evidence that loop diuretics by themselves have albuminuria-reducing effects. Amiloride can reduce albuminuria by inhibiting distal renal tubular epithelial sodium channels, and one study showed a significant reduction in UACR in patients with DM who were treated with amiloride [79,80]. Acetazolamide, a carbonic anhydrase inhibitor, can reduce glomerular hyperfiltration by activating tubuloglomerular feedback [81]. Acetazolamide has been shown to improve transient albuminuria in patients with acute mountain sickness [82].

Although the antialbuminuric effects of most diuretics and their mechanisms require more research to elucidate and the fluid and electrolyte disturbances caused by them are nonnegligible side effects, diuretics should be considered an essential component of combination therapy.

### 3.6. SGLT2 Inhibitors

RAAS inhibitors were the only approved pharmacotherapy for patients with DKD until the advent of SGLT2 inhibitors. SGLT2 inhibitors are oral hypoglycemic drugs that exert hypoglycemic effects by inhibiting SGLT2, which is a cotransporter located in proximal tubule that transports sodium and glucose in a 1:1 ratio via the sodium-potassium ATPase-mediated sodium concentration gradient [83,84]. Nearly eight years of clinical trials have proven that SGLT2 inhibitors have strong cardiorenal protective effects.

The first major study validating the effects of SGLT2 inhibitors was the Empagliflozin, Cardiovascular Outcomes, and Mortality in Type 2 Diabetes (EMPA-REG) Outcome Trial [85]. This trial was designed to evaluate the cardiovascular safety of empagliflozin in patients with DM with cardiovascular diseases. Aside from showing significantly decreased incidence of cardiovascular events in patients treated with empagliflozin, this study also demonstrated a 39% reduction in the composite events of albuminuria progression, creatinine doubling, renal replacement therapy, and renal death. Subsequently, the Canagliflozin Cardiovascular Assessment Study (CANNAS) was conducted to evaluate another SGLT2 inhibitor, canagliflozin. Compared with the results of the EMPA-REG Outcome Trial, canagliflozin showed a similar improvement in cardiovascular outcomes and kidney disease risk in patients with DM, but it significantly increased amputation and fracture rates in these patients, which has not been reported thus far in the cardiovascular outcomes trials of other SGLT2 inhibitors [86]. The third SGLT2 inhibitor, dapalizine, has also been evaluated for its efficacy in major trials. The Dapagliflozin Effect on Cardiovascular Events-Thrombolysis in Myocardial Infarction 58 (DECLARE-TIMI 58) demonstrated significant cardiorenal protection in the dapagliflozin-treated group compared with the placebo group, although the cohort had a low baseline risk of cardiovascular-related events [87]. Major clinical studies related to SGLT2 inhibitors are summarized in Table 2 [85,86,87,88].

It is worth mentioning that the effects of SGLT2 inhibitors on the risk of AKI in patients with DKD are heterogeneous. The cardiovascular safety data from large clinical studies of SGLT2 inhibitors showed significant differences in the effects of different SGLT2 inhibitors on the risk of AKI [85,86,87]. For example, empagliflozin reduced the incidence of AKI, while patients treated with canagliflozin and dapagliflozin developed AKI and even required hospitalization and dialysis, which may have been related to the differences in chemical structure and pharmacology of different SGLT2 inhibitors [89]. To investigate the effects of different SGLT2 inhibitors on AKI risk, a recent experiment compared the effects of empagliflozin and canagliflozin in a rat model of AKI using SCr and blood urea nitrogen (BUN) as indicators of AKI [90]. The results showed that SCr and BUN were higher in the canagliflozin group than the empagliflozin group. Empagliflozin ameliorated tubular necrosis and inflammation, significantly reduced the expression of kidney injury molecule-1, and restored normal levels of urinary microRNA-26a, while canagliflozin had no significant effect on these parameters. These results support the idea that SGLT2 inhibitors play a compound role in AKI, and clinical studies of SGLT2 inhibitors with AKI risk as the primary endpoint should be conducted in the future.

**Figure 1:**
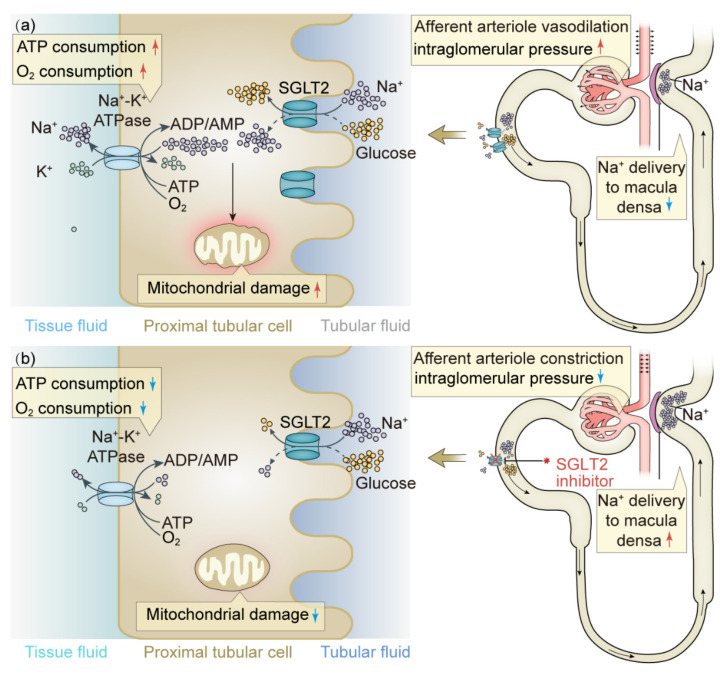
The renoprotective mechanisms of SGLT2 inhibitors. The molecular mechanisms of SGLT2 under hyperglycemia conditions with and without SGLT2 inhibitor. (**a**) SGLT2 is upregulated under hyperglycemic conditions. The reabsorption of sodium and glucose increased, which resulted in the activation of Na^+^-K^+^ ATPase, thus leading to increased ATP and oxygen consumption and mitochondrial damage. The increased resorption of sodium resulted in the low sodium concentration in the macula densa and activated tubuloglomerular feedback, causing the vasodilation of afferent arterioles and increased intraglomerular pressure. (**b**) SGLT2 inhibitors reversed the above changes through inhibiting the resorption of sodium and glucose and increasing the sodium concentration in the macula densa, leading to afferent arteriole constriction and reduced intraglomerular pressure. SGLT2, sodium-dependent glucose transporters 2; ATP, adenosine triphosphate; ADP adenosine diphosphate; AMP, adenosine monophosphate; Na^+^-K^+^ ATPase: sodium-potassium ATPase. Upper arrow in red indicates increase; down arrow in blue indicates decrease.

The physiological mechanisms underlying the cardiorenal benefits of SGLT2 inhibitors have not been thoroughly clarified. It is widely accepted that SGLT2 inhibitors correct afferent arteriole dilatation and glomerular hyperfiltration by increasing the sodium concentration in the macula densa. SGLT2 is upregulated in hyperglycemia and results in increased the reabsorption of sodium in the proximal tubule via the sodium–glucose co-transporter, which in turn leads to the low sodium concentration in the macula densa and activates tubuloglomerular feedback, causing the vasodilation of afferent arterioles and increased intraglomerular pressure and filtration; SGLT2 inhibitors reverse these changes, improve sodium and fluid retention, and reduce intraglomerular pressure [91]. Another hypothesis regarding the renoprotective effect of SGLT2 inhibitors is related to altered renal oxygen consumption [92]. One study showed that the inhibition of SGLT2 reduced oxygen consumption of proximal tubular cells and improved renal cortical oxygenation, mainly by reducing sodium–potassium ATPase activity [93]. In addition, one animal experiment showed that SGLT2 inhibitors can inhibit renal inflammation and fibrosis, and the mechanism may be that a reduction in glucose entering proximal tubule cells can alleviate mitochondrial damage [94]. The renoprotective mechanisms of SGLT2 inhibitors are shown in Figure 1.

Admittedly, SGLT2 inhibitors are limited by potential side effects such as ketoacidosis and urinary tract infections [95]. This may be due to the high doses of SGLT2 inhibitors currently in use; thus, reducing the doses of SGLT2 inhibitors may overcome these challenges. One preclinical study compared the cardiorenal effects of the SGLT-2 inhibitor empagliflozin with telmisartan in nephrectomized rats on a high-salt diet. The effects of 0.6 mg/kg empagliflozin (which was much lower than the dose used in other preclinical studies) on blood pressure and cardiac and renal fibrosis were comparable with the usual dose of telmisartan (5 mg/kg) [96]. Based on this result, future clinical studies should explore the minimum protective dose of SGLT2 inhibitors that can be used without affecting the efficacy of the current standard doses of SGLT2 inhibitors in patients with DKD, which could improve safety and lead to more widespread application of SGLT2 inhibitors.

### 3.7. Incretin Mimetics

Incretins are peptides that are secreted by intestinal cells and are involved in glycemic regulation. Incretins exert hypoglycemic effects by stimulating pancreatic β-cell proliferation to increase insulin and inhibit glucagon release from pancreatic α-cells [97]. GLP-1 is one of the identified incretins and is secreted by L cells in the intestine [98]. GLP-1 works by activating its receptor GLP-1R and is rapidly degraded by DPP-4 after exerting its effects [99]. Based on the above theory, two hypoglycemic drugs have been developed: GLP-1R agonists and DPP-4 inhibitors, which are also known as incretin mimetics. Recent findings suggest that these two drugs also have direct and indirect benefits on the kidneys of patients with DM.

#### 3.7.1. GLP-1R Agonists

Apart from their hypoglycemic effects, emerging evidence illustrated that GLP-1R agonists may have complex renoprotective effects. GLP-1R agonists can induce natriuresis and diuresis by suppressing the sodium-hydrogen exchanger 3 (NHE3) in the brush border of proximal tubular cells, and they also reduce Ang II levels and thereby inhibit renal RAAS activation [100,101]. These mechanisms may partially explain the hypotensive effects of GLP-1R agonists. Cellular experiments illustrated that GLP-1R agonists improved endothelial function by inhibiting endothelin (ET)-1 [102]. Animal models have proven that GLP-1R agonists can lower blood lipids, especially LDL-C, total cholesterol, and triglycerides, and this effect may be achieved by reducing intestinal chylomicron production and activating brown adipose tissue [103,104]. Furthermore, GLP-1 has been demonstrated to cause decreased food intake and weight loss by increasing satiety and reducing appetite [105]. Together, these findings reveal that GLP-1R agonists are able to improve multiple traditional risk factors associated with DKD progression.

At least six GLP-1R agonists have been tested in clinical research (Table 3) [106,107,108,109,110,111]. Compared with placebo, GLP-1R agonists led to varying degrees of improvement in major adverse cardiovascular events and renal function assessment indicators. These observations verify the effect of GLP-1R agonists on cardiorenal function, but more trials are needed to explore the underlying mechanisms.

A comparison of data on emplagiflozin and liraglutide revealed that SGLT2 inhibitors had a more pronounced benefit on renal endpoints (doubling SCr levels and ESRD progression) than GLP-1R agonists [112]. This difference may be explained by the ability to reduce intraglomerular pressure of GLP-1R inhibitors and SGLT2 inhibitors. SGLT2 inhibitors increase the sodium concentration of the macula densa and reduce intraglomerular pressure through tubuloglomerular feedback [91]. Furthermore, SGLT2 inhibitors prevent the proximal tubule reabsorption of glucose and sodium, resulting in decreased salt and water retention and thereby reducing systemic blood pressure [113]. Together with secondary RAAS inhibition and weight loss, these mechanisms constitute the potent antihypertensive effects and renal benefits of SGLT2 inhibitors [114]. In contrast, the antihypertensive mechanisms of GLP-1, mainly by inhibiting NHE3 and reducing Ang II, are less extensive and effective than that of SGLT2 inhibitors [100,101]. Additionally, similar to empagliflozin, liraglutide reduce all-cause mortality, although this is more attributable to its nonrenal benefits [112]. However, it is amazing that liraglutide shows strong potential for reducing albuminuria without obvious influence on SCr level and ESRD progression [107]. The definite renal efficacy of GLP-1R agonists remains to be explored in future studies with renal events as the primary endpoints.

#### 3.7.2. DPP-4 Inhibitors

To date, several DPP-4 inhibitors have been tested in large clinical studies: sitagliptin, saxagliptin, linagliptin, and alogliptin (Table 4) [115,116,117,118]. The renal excretion of linagliptin is a secondary elimination pathway; thus, linagliptin is the only DPP-4 inhibitor that does not require dose adjustment in patients with DM with impaired renal function. Therefore, linagliptin is currently the most commonly used and valued DPP-4 inhibitor [98]. The most recent clinical study investigating the renoprotective effects of linagliptin in patients with DM is the Cardiovascular and Renal Microvascular Outcomes Study of Linagliptin (CARMELINA), which showed that linagliptin might be effective in patients with very high cardiovascular and renal risks, and it improved the predicted eGFR without affecting heart failure hospitalization rates or the risk of other cardiovascular complications [118].

**Figure 2:**
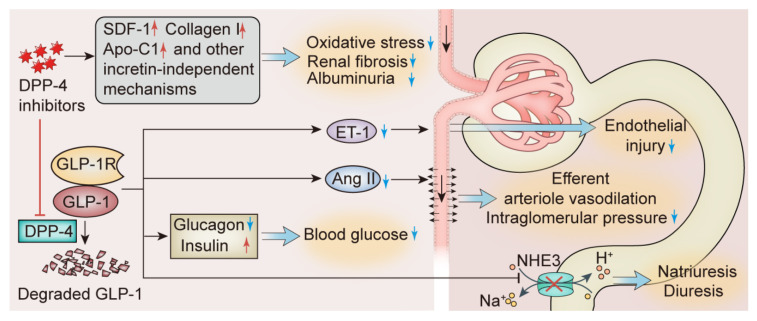
The renoprotective mechanisms of DPP-4 inhibitors. DPP-4 inhibitors can exert renoprotective effects dependent on or independent of the incretin. GLP-1, an identified incretin, works by the activation of GLP-1R. GLP-1 is rapidly degraded by DDP-4 after exerting its effects. After inhibiting the degradation of GLP-1, DPP-4 inhibitors resulted in decreased blood glucose, improved hypertension by inhibiting Ang II, protected endothelial function by inhibiting ET-1, and inhibited NHE3 to exert natriuretic effect. On the other hand, DPP-4 inhibitors inhibited oxidative stress and improved renal fibrosis by regulating SDF-1, collagen I, Apo-C1 and other substrates independent of GLP-1 and its receptors. DPP-4, dipeptidyl peptidase-4; GLP-1, glucagon-like peptide 1; GLP-1R, glucagon-like peptide 1 receptor; SDF-1, stromal cell-derived factor 1; Apo-C1, apolipoprotein C1; Ang II, angiotensin II; ET-1, endothelin-1; NHE3, sodium-hydrogen exchanger 3. Upper arrow in red indicates increase; down arrow in blue indicates decrease; down arrow in black indicates the direction of blood flow.

It was previously thought that the effects of DPP-4 inhibitors were mainly dependent on GLP-1 and its receptor, but the evidence that DPP-4 inhibitors also benefit the kidneys of mice lacking the GLP-1R suggests that the incretin-independent effects of DPP-4 inhibitors need to be explored [119]. Emerging evidence indicates that stromal cell-derived factor 1 (SDF-1), one of the substrates of DPP-4, is upregulated after the inhibition of DPP-4 and may be associated with the antioxidative and antifibrotic effects of DPP-4 inhibitors. [120,121]. One study demonstrated that linagliptin upregulated type I collagen, apolipoprotein C1, and heterogeneous nuclear ribonucleoprotein A2/B1 in nondiabetic rats, and these results were obtained through mass spectrometric analysis [122]. Also using mass spectrometry analysis, another study with GLP-1R knockout mice showed that linagliptin treatment significantly upregulated thymosin b4, downregulated Y-box-binding protein-1, and counteracted nephrectomy-induced transforming growth factor-β1 (TGF-β1) upregulation [123]. These mechanisms may contribute to the antirenal fibrotic effects of DPP-4 inhibitors independent of incretin. The renoprotective mechanisms of DPP-4 inhibitors are shown in Figure 2.

The increased risk of hypoglycemia and reduced insulin clearance make the application of insulin in patients with advanced DKD still controversial [124]. Incretin mimetics have potent hypoglycemic effects with lower risk of hypoglycemia and can be regarded as alternatives for insulin [125]. For obese patients, the combination of GLP-1R agonists and insulin can reduce the risk of weight gain [126]. For patients with cardiovascular risk, GLP-1R agonists have higher safety than insulin [127]. Except saxagliptin, the safety of all DPP-4 inhibitors has been proven in patients with any level of eGFR; therefore, DPP-4 inhibitors may be more suitable than insulin for patients with severe renal insufficiency [124]. Furthermore, since SGLT-2 inhibitors and incretin mimetics lower blood glucose through different mechanisms, the hypoglycemic effects of combination therapy have been studied. One clinical study investigating the effects of triple therapy with SGLT-2 inhibitors, DPP-4 inhibitors, and metformin in patients with T2DM demonstrated that the addition of saxagliptin and dapagliflozin obviously improved metformin-uncontrolled HbA1c [128]. One 28-week clinical trial investigated the effects of exenatide and dapagliflozin, alone and in combination, in patients with T2DM. The results showed that both drugs improved blood glucose that was poorly controlled by metformin, and the combination of the two drugs was more effective than either drug alone [129]. Another 104-week trial came to the similar conclusion that the combination of exenatide and dapagliflozin was beneficial for glycemic control in patients with T2DM and had favorable safety [130]. One recent 16-week clinical trial showed that the addition of SGLT2 inhibitors and GLP-1R agonists while tapering insulin brought greater glycemic improvement without weight gain compared with insulin-only therapy, suggesting that combination therapy may achieve reduction or even withdrawal of insulin [131]. These findings suggest that incretin mimetics and SGLT2 inhibitors have advantages over traditional hypoglycemic drugs in hypoglycemic and renoprotective effects, and with the advent of novel agents, they are expected to be alternative options for patients with DM or DKD.

### 3.8. Endothelin-1 Receptor Antagonists

The ET family was identified in the late 1980s, and three isoforms of ET have been described: ET-1, ET-2, and ET-3 [132]. ET-1 is a strong vasoconstrictor that exerts vasoactive activity mainly by activating ET-A receptors [132].

Preclinical and clinical studies have described that ET-1 activity is increased in patients with DM and that ET-1 receptor antagonists (ERAs) reduce albuminuria and protect renal function [133,134,135]. The renal benefits of ERAs can be partly explained by their hemodynamic effects. The activation of ET-A receptors can constrict efferent arterioles, causing glomerular hypertension, which is associated with water and sodium retention [136]. Therefore, ERAs may improve intraglomerular pressure to preserve renal function and reduce the risk of heart failure due to hypervolemia by inhibiting ET-A receptors. However, the nonhemodynamic renoprotective effects of ERAs are also becoming increasingly valued. For example, the glycocalyx of endothelial cells prevents albumin leakage and regulates vascular homeostasis, and ERAs have been confirmed to protect glycocalyx function in hyperglycemic environments, which may be one of the mechanisms leading to the improvement in albuminuria in patients with DM [137]. Additionally, exposure of protein in cultured podocytes results in ET-1 release, leading to renal injury, and ERAs can preserve podocyte function and reduce the release of ET-1 in the kidney [137]. Moreover, ERAs also exert varying degrees of protection of renal tubular cells and mesangial cells, although whether these effects are directly related to the reduction in albumin leakage remains to be studied [138].

Although available clinical trials have shown that ERAs may be novel drugs for DKD, no consistent conclusions regarding their effectiveness and safety in patients with DKD have been reached. The Avosentan for Overt Diabetic Nephropathy (ASCEND) study showed that patients treated with avosentan demonstrated a reduction in doubled SCr and albuminuria compared with the placebo group, demonstrating that avosentan has renal benefit [134]. Unfortunately, the clinical development of avosentan failed to continue due to major secondary adverse cardiovascular events. Subsequently, another ET-1 antagonist, atrasentan, which is more selective for the ET-A receptor, was evaluated for its safety in the Study of Diabetic Nephropathy with Atrasentan (SONAR) with 2500 subjects [135]. The SONAR study was also terminated early because the primary endpoint was not reached within the expected time, but the results showed that treated patients demonstrated a doubling in SCr and progressed to ESRD at a slower rate. A recent meta-analysis revealed significantly reduced albuminuria and improved eGFR in patients with DKD receiving combined treatment with ERAs and RAAS inhibitors, and decreased blood pressure was also observed. [139]. From these studies, it can be concluded that ERAs may be an effective intervention for controlling blood pressure and albuminuria in patients with DKD with decreased eGFR. However, the presence of side effects, such as heart failure, anemia, and hypoglycemia, is a challenge that needs to be overcome before ERAs can be put into clinical use.

## 4. Surgical Treatment

Metabolic surgery occupies an important position in the interventions of patients with severe obesity with DM; however, emerging evidence implies that metabolic surgery improves the albuminuria and reduces the progression of ESRD in patients with obesity, whereas this renoprotective effect occurs independent of body weight [140,141].

Roux-en-Y gastric bypass (RYGB) is the metabolic surgery with the most prominent renal protective effects. Preclinical studies have demonstrated improved glomerular morphology, reduced mechanical stretch of podocytes, and reduced macrophage infiltration and fibrosis after metabolic surgery [142]. One clinical study demonstrated that RYGB was more effective than drug therapy in relieving albuminuria over 24 months [143]. Transmission electron microscopy showed that RYGB improved the glomerular ultrastructure in rats with DKD [142]. Bulk RNA sequencing showed that RYGB corrected multiple mechanisms related to the pathogenesis of DKD, such as renal fibrosis, inflammation, and biooxidation [144]. In addition, sleeve gastrectomy and duodenojejunal bypass have also been shown to slow the progression of albuminuria, albeit to a lesser extent than RYGB [145,146]. These findings support the growing interest of investigators in metabolic surgery as a potential treatment for decreased renal function in patients with DKD.

Because the clinical specimens of kidneys from patients with DM receiving metabolic surgery are not easily available, the molecular mechanism of its renoprotective effects has not been fully clarified. Additionally, no studies have examined changes in dyslipidemia, blood pressure, and eGFR before and after metabolic surgery, and these deficiencies should be the focus of future research in this field.

## 5. New Potential Therapeutic Strategies

### 5.1. Protein Kinase C Inhibition

Protein kinase C (PKC) is a key intracellular signaling molecule involved in the pathogenesis of DKD. DM can activate PKC through glucose itself, Ang II, and advanced glycation end products (AGEs), and elevated PKC can in turn participate in the pathophysiology of DKD [147]. The effects of the α and β isoforms of PKC have been established in preclinical trials. PKC-α contributes to albuminuria by downregulating proteoglycans on the glomerular basement membrane and modulating vascular endothelial growth factor expression, while PKC-β is involved in the occurrence of vascular dysfunction in DM [148,149].

The PKC-β inhibitor ruboxistaurin is renoprotective according to preclinical and clinical studies. In animal experiment, ruboxistaurin ameliorated the increase of albuminuria and SCr in rats with DKD [150]. In a clinical trial, ruboxistaurin reduced albuminuria and improved eGFR in patients with T2DM [151]. However, large clinical studies are required before the drug can enter the clinic. In addition, investigating the relationship between diabetic complications and other PKC subtypes, such as δ and ε, may provide a novel direction for DKD treatment.

### 5.2. Adiponectin

As an adipokine, adiponectin improves insulin sensitivity and reduces serum glucose levels in obesity-related metabolic disorders, including DM, by promoting glucose transporter-4-mediated glucose uptake in muscle and adipose tissue and inhibiting hepatic gluconeogenesis [152]. In an animal experiment, adiponectin knockout mice exhibited marked albuminuria, and the loss of podocyte foot processes was observed in kidney tissue; adiponectin reversed these changes [153]. Adiponectin has been confirmed to prevent renal hypertrophy in patients with DKD and the mechanism may be related to its promotion of calorie consumption [154,155]. One recently developed adiponectin receptor agonist, AdipoRon, may have better efficacy than adiponectin, but its safety remains to be confirmed [156]. The prospect of adiponectin in the treatment of DKD may be realized in the near future.

### 5.3. Anti-Inflammatory Treatments

DKD is generally considered a noninflammatory disease. However, a genome-wide transcriptome profiling study identified strong inflammatory signaling pathways in the pathogenesis of DKD [157]. This finding is also supported by recent single-nucleus RNA sequencing of renal biopsy specimens from patients with T2DM [158]. Multiple reports have indicated that inflammatory cells, such as leukocytes, monocytes, and macrophages, are associated with the development of DKD, and inflammatory factors such as interleukins (ILs) and tumor necrosis factor-α (TNF-α) in the kidneys of animals and humans with DM are upregulated [159,160]. In addition, one animal study has demonstrated a protective effect of inhibiting the entry of inflammatory cells into the kidneys [161]. These conclusions suggest that inflammation in the kidney is a key pathophysiological basis for the progression of DKD and reveal the potential of targeted anti-inflammatory therapy in the treatment for DKD.

#### 5.3.1. Agents Inhibiting Inflammatory Factors

The proinflammatory factor IL-20 is recognized as a causative factor in kidney injury and may be an important therapeutic target for slowing the progression of DKD [162]. The use of anti-IL-20 monoclonal antibodies has shown renal protection in mouse models, and subsequently, a human recombinant monoclonal antibody against IL-20 demonstrated safety in human experiments [163]. Another IL, IL-1, is related to an increased rate of ESRD [164]. Canakinumab, an IL-1 monoclonal antibody, can decrease the occurrence of myocardial infarction and systemic inflammation [165]; however, whether it has a protective effect on the progression of DKD needs to be verified by including more patients at risk of DKD.

Chemokines and chemokine receptors also play important roles in the progression of DKD. C-C motif chemokine ligand 2 (CCL2), a monocyte chemoattractant protein, has been identified to be overexpressed in kidneys of animals and patients with DM, and inhibition of CCL2 is associated with renoprotective effects [166,167]. Emapticap pegol (NOX-E36), which specifically binds and inhibits CCL2, was demonstrated to have renoprotective effects in a study in patients with T2DM [168]. CCX140-B, a selective CCL2 receptor2 antagonist, has been confirmed to sustainedly improve albuminuria in patients with T2DM in a 52-week phase 2 trial [169].

#### 5.3.2. JAK/STAT Inhibitors

The Janus kinase-signal transducer and activator of transcription (JAK-STAT) pathway is a family of intracellular signaling molecules activated by binding extracellular cytokine, chemokine, growth factor, and hormone ligands to glomerular cell surface receptors, and their persistent activation is closely related to many inflammatory diseases [170]. The analysis of RNA transcription profiles has revealed that the JAK–STAT pathway is significantly upregulated in the kidneys of patients with DKD, especially JAK1 and STAT1, 3, and 5 [171].

Baricitinib is a selective inhibitor of JAK-1 and JAK-2. One clinical study designed to investigate the effects of baricitinib in patients with T2DM showed that treatment with baricitinib in combination with RAAS inhibitors improved albuminuria and reduced inflammatory biomarkers, such as serum TNF receptor 1, TNF receptor 2, and urinary CCL2 [172]. However, adverse events, such as anemia and elevated alanine aminotransferase, may limit its development.

#### 5.3.3. ASK1 Inhibitors

Apoptotic signal-regulated kinase 1 (ASK1) is a stress-responsive mitogen-activated protein kinase (MAPK) that signals through a series of downstream kinases to regulate the expression of target genes, including inflammatory cytokine genes [173]. Kidney biopsy samples have revealed elevated ASK1 activity in patients with DKD. One preclinical experiment has demonstrated that the inhibition of ASK1 could improve eGFR and albuminuria and alleviate histopathological damages in DKD [174].

Selonsertib is a selective ASK1 inhibitor and its renoprotective effect in patients with T2DM was investigated in a clinical study with change of eGFR as the primary endpoint; although selonsertib showed no significant effect on eGFR and UACR compared with placebo, the post hoc analysis suggested that selonsertib had potential renal benefits [175].

### 5.4. Antioxidant Treatments

Oxidative stress caused by the formation of reactive oxygen species (ROS) due to cellular respiratory dysfunction under diabetic conditions is thought to exert a crucial effect in the development of DKD [176]. Hyperglycemia has been demonstrated to promote the overproduction of ROS through a variety of molecular mechanisms, such as the formation of AGEs, activation of PKC, upregulation of the hexosamine pathway, and autoxidation of glucose [177]. Elevated ROS can in turn act as second messengers to redox-modify multiple proteins, including PKC, IκB kinase β, and Kelch-like ECH-associated protein 1 (Keap1), as well as activating alternative downstream signaling pathways that play critical roles in β-cell dysfunction and insulin resistance, facilitating the development of DM and its complications [178,179]. Therefore, although there is insufficient clinical evidence, antioxidant therapy targeting oxidative stress and its downstream targets may achieve precise intervention in patients with DKD.

#### 5.4.1. Nrf2 Activators

Nuclear factor erythroid 2-related factor 2 (Nrf2) is a transcription factor that was confirmed to prevent damage from oxidative stress [180]. Under stress-free conditions, Nrf2 is generally degraded by Keap1-mediated proteasome. When under oxidative stress, the affinity of Keap1 for Nrf2 will decrease, thereby preventing the degradation of Nrf2, and undegraded Nrf2 enters the nucleus to enhance the expression of genes associated with antioxidation through binding to antioxidant response elements (AREs); thus, Nrf2 inhibits oxidative stress mainly through the Nrf2/Keap1/ARE pathway [181].

The most promising Nrf2 agonist at present is bardoxolone methyl, a synthetic derivative of oleanolic acid. One early clinical trial with patients with T2DM designed to investigate the efficiency and safety of bardoxolone methyl demonstrated robust increases in eGFR in subjects, but the trial was terminated prematurely due to the risk of cardiorenal events [182]. In the subsequent Randomized Clinical Trial on the Effect of Bardoxolone Methyl on GFR in Diabetic Kidney Disease Patients (TSUBAKI), it was also confirmed that patients treated with bardoxolone methyl demonstrated recovery in eGFR and had a lower incidence of ESRD [183]. These two trials showed the feasibility of alleviating DKD by modulating the Nrf2/Keap1/ARE pathway, and the ongoing AYAME phase III clinical trial may illustrate this more clearly in the future [184].

#### 5.4.2. NOX Inhibitors

NADPH oxidase (NOX) is an enzyme family proven to be an important source of ROS that is upregulated in patients with DM [185]. NOX4, an isoform of this family, is the enzyme that plays crucial roles in the production of ROS in the diabetic kidney [186]. GKT137831 is an inhibitor that selectively inhibits NOX1/4, and it has been shown to improve the histological damage in mice with DKD [187]. However, GKT137831 did not reduce albuminuria in patients with DM in previous clinical evaluations, and its long-term efficacy in DKD will be further illustrated in an ongoing trial [188]. Recently, another isoform, NOX5, has also demonstrated involvement in the pathogenesis of DKD [189]. Although research on the NOX family is not sufficiently deep, the existing evidence proves that this is a promising therapeutic target for DKD.

#### 5.4.3. Bioactive Antioxidants

Studies have shown that some bioactive compounds with antioxidant properties, either from the diet or from plants, protect against DM and its complications, and these natural products are generally considered to have better safety profiles than other drugs.

Several vitamins are effective antioxidants and are closely linked to the onset of DM. Vitamin C can directly scavenge ROS, and its effect on T2DM has been evaluated in numerous studies. Both animal models and clinical studies have shown that vitamin C significantly inhibits oxidative stress and improves albuminuria and glomerulosclerosis [190,191]. Studies of high doses of vitamin D administered orally over a short period of time have also revealed that vitamin D prevents oxidative stress in macromolecules and balances mitochondrial activity [192,193]. Vitamin E is the most effective antioxidant, especially for protecting lipids from oxidation, and in vivo studies in rats have shown that vitamin E can scavenge free radicals produced by NADPH oxidase [194].

Curcumin, which is found in turmeric, has antioxidant and anti-inflammatory properties [195]. One animal experiment showed that taking curcumin can effectively improve kidney damage and significantly reduce blood glucose concentration [196]. However, the antioxidant mechanism of curcumin is still unclear. In addition to scavenging ROS directly, curcumin may also regulate certain signaling pathways, such as reducing superoxide production by inhibiting the PKC/MAPK signaling pathway and inhibiting Nrf2 degradation by binding to Keap1 [197,198].

Resveratrol is a natural polyphenolic compound that is commonly found in peanuts and berries [199]. Resveratrol has natural antioxidant properties and is a powerful scavenger of superoxide, hydroxyl radicals, and peroxynitrite [200]. The protective effect of resveratrol on cardiac function in hyperglycemic environments has been verified [152]. One animal study demonstrated that resveratrol improved podocyte injury in mice with DM by attenuating oxidative stress, suggesting that resveratrol also had nephroprotective effects [201]. In addition, resveratrol also has a protective effect on diabetic kidneys by activating Sirtuin 1, which is one of the pieces of evidence suggesting that resveratrol may be a new preventative for DKD [202].

### 5.5. Antifibrotic Treatments

Renal fibrosis is the final common pathway of pathophysiological processes such as abnormal renal hemodynamics, a high-glucose environment, oxidative stress, RAAS hyperactivation, ischemia, and inflammation, and it is the main reason for the progression of DKD and the occurrence of ESRD. Some of the therapeutic strategies mentioned above, such as RAAS inhibition, reduce renal fibrosis but with limited efficacy [53]. To achieve more precise interventions, a rational approach may be to develop specific antifibrotic treatments.

Pirfenidone, which is an antifibrotic drug used to treat idiopathic pulmonary fibrosis, was recently also evaluated for its antifibrotic effects in DKD. An animal model suggested that pirfenidone reduced mesangial matrix expansion associated with renal fibrosis but had no effect on albuminuria [203]. One randomized clinical trial showed that low-dose pirfenidone reduced the decline in eGFR in patients with DKD, but gastrointestinal side effects caused by high-dose pirfenidone led to study termination [204]. Pentoxifylline is another promising antifibrotic drug that was previously mainly used to treat vascular diseases and has been proven to attenuate the pathogenesis of renal fibrosis by inhibiting ECM accumulation and cellular proliferation [205]. One clinical trial in patients with T2DM showed that the combination of RAAS inhibitors and pentoxifylline improved eGFR and albuminuria [206].

TGF-β is thought to be a key driver of renal fibrosis in many progressive kidney diseases because of its ability to lead to myofibroblast activation and the overproduction of ECM [207]. Therefore, drugs targeting TGF-β are the current focus in the field of antifibrosis research. Tranilast, a TGF-β blocker, showed benefits in patients with DKD in several small clinical studies [208,209]. Subsequently, a derivative of tranilast, FT011, was developed that was shown to attenuate glomerulosclerosis and albuminuria in rats with DKD, but it has not been clinically evaluated [210]. Anti-TGF-β1 monoclonal antibodies were tested in a phase II clinical trial, but no obvious efficacy was observed [211]. Another method of blocking TGF-β1 is to inhibit connective tissue growth factor (CTGF), which is a downstream molecule of TGF-β. An animal study demonstrated that the upregulation of CTGF expression was involved in the progression of DKD [212]. One phase I study demonstrated that anti-CTGF monoclonal antibodies reduced albuminuria in patients with DM, but their efficacy needs to be verified in larger experiments [213]. Targeting cell division autoantigen 1 (CDA1) is thought to suppress the effect of TGF-β in renal fibrosis. CDA1BP1 is a protein identified as a key regulator of CDA1 activity [214]. A recent preclinical experiment demonstrated that inhibitors targeting the CDA1/CDA1BP1 axis can significantly attenuate renal ECM accumulation and glomerular injury, and clinical trials will follow [215].

Because TGF-β has important immunomodulatory effects and it is difficult to directly target TGF-β, many studies have been devoted to finding new antifibrotic targets and drugs in recent years.

Deficiencies in nitric oxide (NO) and cyclic guanosine monophosphate (cGMP) have been shown to directly promote the progression of renal fibrosis [216]. Soluble guanylate cyclase (sGC) regulates NO signaling by catalyzing the formation of cGMP, and the prosthetic heme moiety of its β subunit is integral for NO binding [217]. Two drugs, sGC stimulators and sGC activators, have been developed to promote the sGC-catalyzed production of cGMP and NO through different mechanisms [218]. The sGC stimulators directly stimulate sGC and stabilize the nitroso-heme complex of sGC to synergize with NO [219]. The earliest reported sGC stimulator is YC-1, but its potency and specificity are weak [220]. The more potent sGC stimulators BAY 41-2272 and BAY 41-8543 were proven to reduce albuminuria and prevent renal matrix deposition in rat models [221,222]. Riociguat is the first approved sGC stimulator. In preclinical trials, riociguat treatment improved glomerulosclerosis and interstitial fibrosis in hypertensive rats, and the combined treatment with riociguat and telmisartan reduced albuminuria in mice with DM [223,224]. Unlike sGC stimulators, sGC activators increase the active iron of sGC only when heme iron is oxidized, and they exert only an additive effect with NO through binding to the heme-binding complex [219]. BAY 58-2667, a sGC activator, was shown to ameliorate podocyte damage in rats with DM and improve eGFR and renal fibrosis in mice with DM [225,226]. Another sGC activator, BI 703404, was proven to reduce albuminuria and glomerulosclerosis in rats with DKD [227].

Hypoxia-inducible factor (HIF) is a nuclear transcription factor activated under hypoxic conditions, consisting of HIF-α and HIF-1β in the form of heterodimers [228]. HIF-α has three isoforms, among which HIF-1α mainly regulated by oxygen and is normally hydroxylated by prolyl hydroxylase domain protein (PHD), after which it is then degraded by von Hippel–Lindau protein (VHL) [229]. Hypoxia and the aberrant activation of HIF signaling have been identified as contributing factors for renal fibrosis in patients with DM, and HIF stabilizers that inhibit the degradation of HIF-α may be promising for preventing renal fibrosis, which has been confirmed in preclinical studies [230]. Cobalt chloride ameliorated renal injury in rats with DM by inhibiting the binding of HIF-1α to VHL [231]. The PHD inhibitor enarodustat, which is primarily used to treat renal anemia, has recently been indicated to be potentially renoprotective in mice with DM, and more HIF stabilators are being actively developed [232].

Apelin, which is an adipokine found in adipose tissue, has been shown to alleviate clinical symptoms in patients with DM by regulating blood glucose [233]. In the field of DKD, a study reported that Apelin-13 can inhibit the epithelial mesenchymal transition of glomerular cells in a high-glucose environment, ultimately delaying the occurrence of DKD [234]. One recent animal experiment showed that Apelin-13 treatment alleviated DKD by inhibiting glomerular fibrosis [235].

Pyrroloquinoline quinone (PQQ), which is a natural bioactive compound, was previously shown to protect human renal tubular epithelial cells under high-glucose conditions, but the underlying mechanism is unclear [236]. In a recent study, the protective effect of PQQ on DKD-induced renal fibrosis was assessed by inhibiting the pyroptosis signaling pathway in mice with T1DM, and it was concluded that PQQ could alleviate renal fibrosis by inhibiting the activation of the nuclear factor-κB/pyroptosis pathway under hyperglycemic conditions [237].

SIRT3, which is a major mitochondrial deacetylase, blocks organ fibrosis by modulating TGF-β/Smad signaling [238]. One study showed that inhibiting SIRT3 by administering SIRT3 siRNA in diabetic mice induces renal fibrosis, further indicating that the mechanism is that SIRT3 deficiency induces abnormal glycolysis, which can promote fibrotic programming [239]. This suggests that the restoration of SIRT3 may be an effective strategy for combatting diabetes-related renal fibrosis by inhibiting abnormal glycolysis.

Although most of these treatments have not been clinically evaluated, their potential to inhibit diabetes-related renal fibrosis has brought renewed enthusiasm to the field of DKD therapeutics.

### 5.6. Treatments Targeting Autophagy

Autophagy is a highly conserved lysosomal degradation pathway that clears damaged proteins and organelles to maintain cellular homeostasis [240]. Autophagy is demonstrated to be deregulated by RAAS activation, insulin resistance, and oxidative stress-induced dysregulation under diabetic conditions, resulting in glomerular and tubulointerstitial lesions [241]. One animal study demonstrated that the removal of autophagy-related gene 5 from the renal proximal tubule led to autophagy deficits and more severe renal impairment, suggesting that autophagy has a protective effect in DKD [242]. Therefore, autophagy-mediated pathway upregulation may be a useful target for DKD treatment.

The mammalian target of rapamycin (mTOR) signaling pathway is an important element of autophagy, and an animal model showed that rapamycin has beneficial effects on the histopathological changes in rats with DKD by inhibiting the mTOR signaling pathway, but it has some side effects, including immunosuppression and renal toxicity [243,244]. Therefore, it is necessary to identify safer mTOR inhibitors. Emerging evidence shows that Chinese medicines may have unique potential in this regard. Cyclocarya paliurus, which is a Chinese herb, has been confirmed to reduce albuminuria and SCr levels, as well as improving mesangial matrix deposition and glomerular fibrosis by reducing mTOR phosphorylation via the AMP-activated protein kinase-mTOR-regulated autophagy pathway in a DKD rat model [245]. Another traditional Chinese medicine, Jiedu Tongluo Baoshen Formula, can downregulate the expression of renal mTOR-related proteins in rats with DM, enhancing podocyte autophagy, reducing podocyte damage, and effectively treating DKD [246].

Identifying other autophagy-related signaling pathways is also a potential renoprotective approach for future studies. One recent study demonstrated that hyperglycemia increased the expression of microRNA-214 (miR-214) in the kidney, which in turn led to the downregulation of unc-51-like autophagy-activating kinase 1 (ULK1), which may be responsible for the autophagy damage in DM. Removing the miR-214 of renal proximal tubules reduced the downregulation of ULK1 in kidneys and prevented autophagy damage, whereas p53 inhibition decreased the induction of miR-214 and ameliorated renal injury [247]. This experiment identified the role of p53/miR-214/ULK1 pathway in renal autophagy damage, which is expected to be a possible therapeutic target for DKD.

## 6. Interventions for Nonalbuminuric DKD

DKD was previously thought to have a uniform natural history characterized by the development of persistent albuminuria with a decline in eGFR and eventually leading to renal failure [248]. However, cross-sectional studies have shown that there are 20% and 40% of patients with T1DM and T2DM respectively who develop DKD without albuminuria, and this phenotype is described as nonalbuminuric DKD [249,250]. Nonalbuminuric DKD is significantly different from albuminuric DKD in phenotype and outcomes, and the treatment for these patients should be more individualized [251]. Although there is no specific intervention, studies for the pathogenesis and characteristics of nonalbuminuric DKD may provide theoretical guidance for its preventions and treatments.

The heterogeneity of nonalbuminuric DKD makes its diagnosis crucial and challenging. UACR is generally considered to be an important criterion for early diagnosis of DKD, but for patients without albuminuria, eGFR is clearly a more appropriate diagnostic indicator [252]. A variety of biomarkers, such as NGAL, liver-type fatty acid-binding protein, and lipocalin-type prostaglandin D2 synthase, have been demonstrated to evaluate the progression of nonalbuminuric DKD [253,254,255]. Furthermore, albuminuric DKD is closely associated with microvascular diseases, but the findings of carotid artery injury and intrarenal arteriosclerosis suggest macrovascular dysfunctions may play a more critical role in the progression of nonalbuminuric DKD [256]. The histopathology suggested that renal interstitial fibrosis, which leads to declining eGFR independent of albuminuria, was more severe in patients with nonalbuminuric DKD than in those with albuminuria [257]. These facts suggest that macrovascular and renal interstitial damage may also serve as predictors of the development of nonalbuminuric DKD.

One meta-analysis demonstrated that renal function declined more rapidly in men than in women amo patients with kidney diseases caused by other causes, but it was opposite in populations with nonalbuminuric DKD [258]. One clinical study showed that the sample of patients with nonalbuminuric DKD had a much higher percentage of Caucasians than Asians [259]. Compared with patients with albuminuric DKD, patients with nonalbuminuric DKD have higher body mass index and LDL [260]. These differences in incidence reveal the high-risk groups for nonalbuminuric DKD, and measures to control risk factors, such as weight loss and lipid-lowering therapies, may be beneficial for preventing the occurrence of nonalbuminuric DKD.

Pharmacological therapies for nonalbuminuric DKD should focus on improving eGFR rather than traditional antialbuminuric therapy. Many patients with nonalbuminuric DKD have a usage history of RAAS inhibitors, and the discontinuation of RAAS inhibitors often leads to the appearance of proteinuria [257]. The clinical outcomes of these patients demonstrated that RAAS inhibitors could reduce albuminuria, but they failed to inhibit the decline in eGFR [249]. One recent clinical trial showed that ertugliflozin improved the declining eGFR in patients with DKD, especially those without albuminuria, suggesting that SGLT2 inhibitors may be more suitable for patients with nonalbuminuric DKD [261]. More clinical studies targeting nonalbuminuric patients should be the focus of future research.

## 7. Conclusions

In recent years, the adverse effects of DKD in patients with DM have received increasing attention, and the importance of developing new treatments for DKD has been widely recognized. The demonstration of the renoprotective effects of SGLT2 inhibitors, GLP-1R agonists, and DPP-4 inhibitors has introduced a new era in the field of DKD treatment. Novel drugs targeting inflammation, fibrosis, oxidative stress, and other new targets also show promise for patients with DKD, although a large number of clinical studies are needed to assess their safety and efficacy. In addition, a single class of drug therapy may not produce significant therapeutic effects, but with the continued emergence of agents that work through different mechanisms, combination therapy with multiple drugs is expected to achieve a breakthrough in the treatment of DKD in the future.

## Figures and Tables

**Table 1:** Major clinical studies related to aldosterone antagonists.

Drugs	Clinical Trials	Study Design	Number of Patients	Primary Outcomes	Secondary Outcomes	Safety Signal	Reference
Eplerenone	Eplerenone in Mild Patients Hospitalization and Survival Study in Heart Failure (EMPHASIS-HF) study	Multicenter, randomized, double-blind, placebo-controlled trial of eplerenone 25–50 mg qd in patients who aged at least 55 years with NYHA functional class II symptoms and an ejection fraction ≤ 35% (median 21 months).	2737	37% reduction in MACE (death from CV causes or hospitalization for HF)	24% reduction in death from CV causes; 42% reduction in hospitalization for HF; 31% reduction in hospitalization for CV causes; no effect on hospitalization for worsening renal function; no effect on renal failure.	Increased risk of hyperkalemia (11.8% vs. 7.2%, *p* < 0.001) in group with eplerenone compared with placebo.	[63]
Finerenone	Chronic Kidney Disease Outcomes in Type 2 Diabetes (FIDELIO) study	Multicenter, randomized, double-blind, placebo-controlled trial of finerenone 10–20 mg qd in patients with T2DM who had persistent, moderately elevated albuminuria with an eGFR of 25 to < 60 mL/minute/1.73 m^2^ or persistent, severely elevated albuminuria with an eGFR of 25 to <75 mL/minute/1.73 m^2^ (median 2.6 years).	5734	18% reduction in composite renal outcome (kidney failure, a sustained decrease of at least 40% in the eGFR from baseline, or death from renal causes); 13% reduction in kidney failure (ESRD or sustained decrease in eGFR to <15 mL/min/1.73 m^2^); 19% reduction in sustained decrease of ≥40% in eGFR from baseline.	14% reduction in key secondary composite outcome (the death from CV causes, nonfatal MI, nonfatal stroke, or hospitalization for HF).	The rate of hyperkalemia and hyperkalemia-related discontinuation for patients with finerenone vs. placebo were 18.3% vs. 9.0% and 2.3% vs. 0.9%.	[67]
	Cardiovascular Events with Finerenone in Kidney Disease and Type 2 Diabetes (FIGARO) study	Multicenter, randomized, double-blind, placebo-controlled trial of finerenone 10–20 mg qd in patients with T2DM who had persistent, moderately elevated albuminuria with an eGFR of 25 to 90 mL/minute/1.73 m^2^ or persistent, severely elevated albuminuria and an eGFR of at least 60 mL/minute/1.73 m^2^ (median 3.4 years).	7437	13% reduction in MACE (the death from CV causes, nonfatal MI, nonfatal stroke, or hospitalization for HF).	18% reduction in composite renal outcome (kidney failure, a sustained decrease from baseline of at least 40% in the eGFR, or death from renal causes); 29% reduction in kidney failure (ESRD or sustained decrease in eGFR to <15 mL/min/1.73 m^2^); 13% reduction in sustained decrease of ≥40% in eGFR from baseline.	The rate of hyperkalemia, hyperkalemia-related discontinuation and hospitalization for patients with finerenone vs. placebo were 10.8% vs. 5.3%, 1.2% vs. 0.4% and 0.6% vs. 0.1%.	[68]

NYHA, New York Heart Association; MACE, major adverse cardiovascular events; CV, cardiovascular; HF, heart failure; T2DM, type 2 diabetes mellitus; eGFR, estimated glomerular filtration rate; ESRD, end-stage renal disease; MI, myocardial infarction.

**Table 2:** Major clinical studies related to SGLT2 inhibitors.

Drugs	Clinical Trials	Study Design	Number of Patients	Primary Outcomes	Secondary Outcomes	Safety Signals	Reference
Empagliflozin	Empagliflozin, Cardiovascular Outcomes, and Mortality in Type 2 Diabetes (EMPA-REG) Outcome Trial	Multicenter, randomized, double-blind, placebo-controlled trial of empagliflozin 10 mg or 25 mg qw in patients with T2DM who had a BMI of 45 or less and an eGFR of at least 30 mL/min/1.73 m^2^ (median 3.1 years).	7020	14% reduction in MACE (the death from CV causes, nonfatal MI, or nonfatal stroke).	11% reduction in key secondary composite outcome (MACE or hospitalization for UA); 39% reduction in composite renal outcome (onset of macroalbuminuria, doubling of the SCr and an eGFR of ≤45 mL/min/1.73 m^2^, the need for renal replacement therapy, or death from renal disease).	Increased risk of genital infection in group with 10 mg empagliflozin (6.5% vs. 1.8%, *p* < 0.001) and group with 25 mg empagliflozin (6.3% vs. 1.8%, *p* < 0.001) compared with placebo.	[85]
Canagliflozin	Canagliflozin Cardiovascular Assessment Study (CANVAS)	Multicenter, randomized, double-blind, placebo-controlled trial of canagliflozin 100 mg or 300 mg qd in patients with T2DM who had a history of symptomatic atherosclerotic CV disease or were 50 years of age or older with at least 2 risk factors for CV disease (median 188.2 weeks).	10,142	14% reduction in the death from CV causes, nonfatal MI, or nonfatal stroke.	70% increase in regression of albuminuria; 40% reduction in composite renal outcome (40% reduction in eGFR, the need for renal replacement therapy, or death from renal disease).	Increased risk of genital infection (0.35% vs. 0.11%, *p* < 0.001) and increased risk of amputation of toes, feet, or legs (0.63% vs. 0.34%, *p* < 0.001) in group with canagliflozin compared with placebo.	[86]
Dapagliflozin	Dapagliflozin Effect on Cardiovascular Events-Thrombolysis in Myocardial Infarction 58 (DECLARE-TIMI 58) trial	Multicenter, randomized, double-blind, placebo-controlled trial of dapagliflozin 10 mg qd patients with T2DM who had or were at risk for atherosclerotic CV disease and had a HbA1c ≥ 6.5% but <12.0%, and a Scr clearance ≥ 60 mL/min (median 4.2 years).	17,160	No effect on MACE (the death from CV causes, nonfatal MI, or nonfatal stroke); 17% reduction in CV death or hospitalization for heart failure.	24% reduction in composite renal outcome (new ESRD, ≥40% decrease in eGFR to <60, death from renal or CV causes); No effect on secondary efficacy outcomes (the death from any cause).	Increased risk of genital infection (0.9% vs. 0.1%, *p* < 0.001) in group with dapagliflozin compared with placebo.	[87]
Canagliflozin	Canagliflozin and Renal Endpoints in Diabetes with Established Nephropathy Clinical Evaluation (CREDENCE) trial	Multicenter, randomized, double-blind, placebo-controlled trial of canagliflozin 100 mg qd in patients with T2DM who had an eGFR of 30 to <90 mL/min/1.73 m^2^ and albuminuria and were treated with RAAS inhibitors (median 2.62 years).	4401	32% reduction in ESRD (the need for renal replacement therapy or sustained eGFR of <15 mL/min/1.73 m^2^); 34% reduction in composite renal outcome (ESRD, doubling of SCr, or death from renal disease).	20% reduction in MACE (the death from CV causes, nonfatal MI, or nonfatal stroke).	No significant difference in rates of adverse events (amputation, fracture, and diabetic ketoacidosis) between two groups.	[88]

SGLT2, sodium-dependent glucose transporters 2; T2DM, type 2 diabetes mellitus; BMI, body mass index; eGFR, estimated glomerular filtration rate; MACE, major adverse cardiovascular events; CV, cardiovascular; MI, myocardial infarction; UA, unstable angina; SCr, serum creatinine; HbA1c, glycosylated hemoglobin; ESRD, end-stage renal disease.

**Table 3:** Major clinical studies related to GLP-1R agonists.

Drugs	Clinical Trials	Study Design	Number of Patients	Primary Outcomes	Secondary Outcomes	Safety Signals	Reference
Lixisenatide	Lixisenatide in Patients with Type 2 Diabetes and Acute Coronary Syndrome (ELIXA) trial	Multicenter, randomized, double-blind, placebo-controlled trial of lixisenatide 10–20 μg qd in patients with T2DM who had a MI or had been hospitalized for UA within the previous 180 days (median 25 months).	6068	No effect on MACE (the death from CV causes, nonfatal MI, or nonfatal stroke).	19.2% reduction in new onset macroalbuminuria; No significant effect on eGFR	Increased risk of gastrointestinal event (4.9% vs. 1.2%, *p* < 0.001) in group with lixisenatide compared with placebo.	[106]
Liraglutide	Liraglutide and Cardiovascular Outcomes in Type 2 Diabetes (LEADER) study	Multicenter, randomized, double-blind, placebo-controlled trial of liraglutide 1.8 mg qd in patients with T2DM who had a HbA1c ≥ 7.0% and aged at least 50 years old with at least one CV coexisting condition or aged at least 60 years with at least one CV risk factor (median 3.8 years).	9340	13% reduction in MACE (the death from CV causes, nonfatal MI, or nonfatal stroke).	22% reduction in composite renal outcome (onset of macroalbuminuria, doubling of the SCr and an eGFR of ≤45 mL/minute/1.73 m^2^, the need for continuous renal replacement therapy, or death from renal disease); 26% reduction in progression of albuminuria	Acute gallstone disease is the main severe adverse event. Nausea, vomiting and diarrhea are the most common causes leading to the discontinuation.	[107]
Semaglutide	Semaglutide and Cardiovascular Outcomes in Patients with Type 2 Diabetes (SUSTAIN-6) trial	Multicenter, randomized, double-blind, placebo-controlled trial of semaglutide 0.5 or 1.0 mg qw in patients with T2DM who had a HbA1c level ≥ 7% and aged at least 50 years or with established CV disease, chronic heart failure or CKD of stage 3, or aged at least with one CV risk factor (median 2.1 years).	3297	26% reduction in MACE (the death from CV causes, nonfatal MI, or nonfatal stroke).	36% reduction in new or worsening nephropathy	Increased risk of gastrointestinal disease and reduced risk of severe adverse events (including serious cardiac disorders) in group with semaglutide compared with placebo.	[108]
Exenatide	Effects of Once-Weekly Exenatide on Cardiovascular Outcomes in Type 2 Diabetes (EXSCEL) trial	Multicenter, randomized, double-blind, placebo-controlled trial of exenatide 2 mg qw in T2DM patients who had HbA1c of 6.5–10.0% and with or without previous CV disease (median 3.2 years).	14,752	9% reduction in MACE (the death from CV causes, nonfatal MI, or nonfatal stroke).	12% reduction in composite renal outcome (onset of macroalbuminuria, doubling of the Scr and an eGFR of ≤45 mL/minute/1.73 m^2^, the need for continuous renal replacement therapy, or death from renal disease); 26% reduction in progression of albuminuria)	No significant difference in risk of adverse events (acute pancreatitis, pancreatic cancer, severe hypoglycemia and medullary thyroid carcinoma) between two groups.	[109]
Albiglutide	Albiglutide and cardiovascular outcomes in patients with type 2 diabetes and cardiovascular disease (HARMONY) trial	Multicenter, randomized, double-blind, placebo-controlled trial of albiglutide 1.5 mg qw in patients aged at least 40 years with T2DM who had HbA1c ≥7.0% and established disease of the coronary, cerebrovascular, or peripheral arterial circulation (median 1.5 years).	9463	22% reduction in MACE (the death from CV causes, nonfatal MI, or nonfatal stroke).	Renal outcomes are not available	No significant difference in risk of adverse events (acute pancreatitis, pancreatic cancer, severe hypoglycemia and medullary thyroid carcinoma) between two groups.	[110]
Dulaglutide	Dulaglutide and cardiovascular outcomes in type 2 diabetes (REWIND) trial	Multicenter, randomized, double-blind, placebo-controlled trial of dulaglutide 1.5 mg qw in patients with T2DM who aged at least 50 years with CV or renal risk factors (median 5.4 years).	9901	22% reduction in MACE (the death from CV causes, nonfatal MI, or nonfatal stroke)	15% reduction in composite renal outcome (development of UACR >33·9 mg/mmol in those with lower baseline concentration, sustained ≥30% decline in eGFR, or the need of renal replacement therapy)	No significant difference in risk of severe adverse events and increased risk in gastrointestinal event (47.4% vs. 37.1%, *p* < 0.0001) in group with dulaglutide compared with placebo.	[111]

GLP-1R, Glucagon-like peptide 1 receptor; T2DM, type 2 diabetes mellitus; MI, myocardial infarction; UA, unstable angina; MACE, major adverse cardiovascular events; CV, cardiovascular; eGFR, estimated glomerular filtration rate; HbA1c, glycosylated hemoglobin; SCr, serum creatinine; CKD, chronic kidney disease; UACR, urinary albumin-to-creatinine ratio.

**Table 4:** Major clinical studies related to DPP-4 inhibitors.

Drugs	Clinical Trials	Study Design	Number of Patients	Primary Outcomes	Secondary Outcomes	Safety Signals	Reference
Saxagliptin	Saxagliptin and Cardiovascular Outcomes in Patients with Type 2 Diabetes Mellitus (SAVOR- TIMI 53) trial	Multicenter, randomized, double-blind, placebo-controlled trial of saxagliptin 5 mg qd in patients with T2DM who had a HbA1c of 6.5–12.0%, and either a history of established CV disease or multiple risk factors for vascular disease (median 2.1 years).	16,492	No effect on MACE (the death from CV causes, nonfatal MI, or nonfatal stroke).	No effect on secondary efficacy end point (MACE plus hospitalization for heart failure, coronary revascularization, or UA); No effect on eGFR.	Increased risk of hypoglycemia (15.3% vs. 13.4%, *p* < 0.001) in group with saxagliptin compared with placebo.	[115]
Alogliptin	Alogliptin after Acute Coronary Syndrome in Patients with Type 2 Diabetes (EXAMINE)	Multicenter, randomized, double-blind, placebo-controlled trial of alogliptin 25 mg/12.5 mg/6.25 mg qd in patients with T2DM and either an acute MI or UA requiring hospitalization within the previous 15 to 90 days (median 18 months).	5380	No effect on MACE (the death from CV causes, nonfatal MI, or nonfatal stroke).	No effect on principal secondary end point (MACE with the addition of urgent revascularization due to UA within 24 h after hospital admission); No effect on eGFR renal replacement therapy	No significant difference in risk of adverse events (severe hypoglycemia, cancer, dialysis, acute pancreatitis, pancreatic cancer and angioedema) between two groups.	[116]
Sitagliptin	Effect of Sitagliptin on Cardiovascular Outcomes in Type 2 Diabetes (TECOS) study	Multicenter, randomized, double-blind, placebo-controlled trial of sitagliptin 100 mg qd in patients with T2DM who had established atherosclerotic CV disease, and HbA1c of 6.5–8.0% and were receiving stable-dose monotherapy or dual-combination therapy of hypoglycemic agent (median 3.0 years).	14,671	No effect on MACE (time to CV death, nonfatal MI, nonfatal stroke, or hospitalization for UA).	no effect on eGFR	No significant difference in risk of adverse events (infections, cancer, renal failure, severe hypoglycemia, acute pancreatitis and pancreatic cancer) between two groups.	[117]
Linagliptin	The Cardiovascular and Renal Microvascular Outcome Study With Linagliptin (CARMELINA)	Multicenter, randomized, double-blind, placebo-controlled trial of linagliptin 5 mg qd in patients with T2DM who had HbA1c of 6.5–10.0%, history of vascular disease and UACR > 200 mg/g, and reduced eGFR and micro- or macroalbuminuria (median 2.2 years).	6991	No effect on MACE (time to first occurrence of the composite of CV death, nonfatal MI, or nonfatal stroke).	No effect on secondary outcome (time to first occurrence of adjudicated death due to renal failure, ESRD, or sustained ≥40% decrease in eGFR from baseline).	The rate of adverse events, hypoglycemia, and acute pancreatitis for patients with linagliptin vs. placebo were 77.2% vs. 78.1%, 29.7% vs. 29.4%, and 0.3% vs. 0.1%.	[118]

DPP-4, dipeptidyl peptidase-4; T2DM, type 2 diabetes mellitus; HbA1c, glycosylated hemoglobin; CV, cardiovascular; MACE, major adverse cardiovascular events; MI, myocardial infarction; UA, unstable angina; eGFR, estimated glomerular filtration rate; UACR, urinary albumin-to-creatinine ratio; ESRD, end-stage renal disease.

## Data Availability

Not applicable.
